# An innovative concept of use of redox-active electrolyte in asymmetric capacitor based on MWCNTs/MnO_2_ and Fe_2_O_3_ thin films

**DOI:** 10.1038/srep39205

**Published:** 2016-12-16

**Authors:** Nilesh R. Chodankar, Deepak P. Dubal, Abhishek C. Lokhande, Amar M. Patil, Jin H. Kim, Chandrakant D. Lokhande

**Affiliations:** 1 Thin Film Physics Laboratory, Department of Physics, Shivaji University, Kolhapur, - 416004 (M.S), India; 2 School of Applied Chemical Engineering, Chonnam National University, Gwangju 500-757, South Korea; 3 Catalan Institute of Nanoscience and Nanotechnology (ICN2), CSIC and The Barcelona Institute of Science and Technology, Campus UAB, Bellaterra, 08193 Barcelona, Spain; 4 Department of Materials Science and Engineering, Chonnam National University, Gwangju 500-757, South Korea; 5 Centre for Interdisciplinary Studies, D.Y. Patil University, Kolhapur, (M.S.) India

## Abstract

In present investigation, we have prepared a nanocomposites of highly porous MnO_2_ spongy balls and multi-walled carbon nanotubes (MWCNTs) in thin film form and tested in novel redox-active electrolyte (K_3_[Fe(CN)_6_] doped aqueous Na_2_SO_4_) for supercapacitor application. Briefly, MWCNTs were deposited on stainless steel substrate by “dip and dry” method followed by electrodeposition of MnO_2_ spongy balls. Further, the supercapacitive properties of these hybrid thin films were evaluated in hybrid electrolyte ((K_3_[Fe(CN)_6_ doped aqueous Na_2_SO_4_). Thus, this is the first proof-of-design where redox-active electrolyte is applied to MWCNTs/MnO_2_ hybrid thin films. Impressively, the MWCNTs/MnO_2_ hybrid film showed a significant improvement in electrochemical performance with maximum specific capacitance of 1012 Fg^−1^ at 2 mA cm^−2^ current density in redox-active electrolyte, which is 1.5-fold higher than that of conventional electrolyte (Na_2_SO_4_). Further, asymmetric capacitor based on MWCNTs/MnO_2_ hybrid film as positive and Fe_2_O_3_ thin film as negative electrode was fabricated and tested in redox-active electrolytes. Strikingly, MWCNTs/MnO_2_//Fe_2_O_3_ asymmetric cell showed an excellent supercapacitive performance with maximum specific capacitance of 226 Fg^−1^ and specific energy of 54.39 Wh kg^−1^ at specific power of 667 Wkg^−1^. Strikingly, actual practical demonstration shows lightning of 567 red LEDs suggesting “ready-to sell” product for industries.

Current environmental issue requires urgent attention to avoid the vulnerable to disasters now and in the future. The best weapon against these environmental consequences is the solicitation of clean energy from the renewable energy resources (such as solar, wind and ocean energies). Not only solicitation but also storing these energies in worthy energy storage devices is emergently postulated. Presently, supercapacitors (SCs) and Li-ion batteries (LIBs) are the two critical energy storage devices; thus, they hold great attention form scientific and technological aspects^1^. In comparison with LIBs, SCs acquire front position in the column of energy storage devices, because of its glorious power density, longer cycle life, safer, requirement of a simple charging circuit, low-cost, and eco-friendly nature^2^. In many advance electronic gadgets, SCs are used as power source in combination with battery/or fuel cell. For example, in advance hybrid electric vehicles, SCs are used with batteries/fuel cells to provide the peak power during acceleration and to retrieve the waste energy during breaking.

In order to explore SCs for advanced electronic applications, there is need to increase energy density of present SCs devices without sacrificing its power density and cycling life^3^,^4^. According to the equation E = 0.5 CV^2^ (where C and V are the specific capacitance and the operating potential window), there are two possible approaches to increase energy density of SCs^5^,^6^,^7^,^8^. Thus, accordingly, the energy density can be increased by increasing capacitance of the electrode material and by extending voltage window of the device^9^,^10^. In last decade, several efforts have been taken to increase the capacitance of electrode materials such as nanostructurization, nanocomposites etc. as well as use of electrolytes which can be operated at high voltages such as organic, ionic liquid electrolytes etc. In addition, the most promising and advance track to improve the energy density of SCs is the widening voltage window of the device by fabricating the asymmetric SCs where two different electrodes materials (different charge storing) in the aqueous electrolyte. Typically, the working voltage limit of the asymmetric SCs device is defined by the electrolyte overpotential and the work function difference of negative and positive electrodes^11^,^12^.

The different redox-active (material with multiple oxidation state) transition metal oxides (TMOs) like MnO_2_, Ni(OH)_2_, WO_3_, V_2_O_5_, CuO, Fe_2_O_3_, MoO_3_ have been utilized for SCs device fabrication^13^,^14^,^15^, but the bounding gene for TMOs is its lower electrical conductivity, cycling stability and power capability. As a result, the hybridization of TMOs with carbon based materials (like MWCNTs) effectively modifies the electrochemical features of TMOs. The MWCNTs in hybrid electrode not only improve the conductivity but also provide the large active surface area for hosting the TMOs/or electrolyte ions for electrochemical reactions^16^,^17^,^18^,^19^.

In present investigation, we proposed a novel triple hybrid system where we designed hybrid electrode (nanostructured nanocomposites), hybrid device (asymmetric device) with hybrid electrolyte (redox-active species doped). This is an excellent proof-of-design where redox-active electrolyte is used in asymmetric capacitors. Firstly, we combine the MWCNTs and MnO_2_ in a single electrode having high power capability and enhanced energy density. The nanostructured MnO_2_ undergoes the redox reactions with protons/or cations of electrolyte, which enhances the specific capacitance of electrode. Further, the electrochemical features of MWCNTs/MnO_2_ thin film have been studied in the aqueous Na_2_SO_4_ and the redox-active electrolyte (K_3_[Fe(CN)_6_] doped aqueous Na_2_SO_4_). Furthermore, the nanostructured Fe_2_O_3_ thin film have been prepared and used as a negative electrode for asymmetric SCs device. The MWCNTs/MnO_2_//Fe_2_O_3_ asymmetric SCs device works at operating potential window of 2.0 V in optimized redox-active electrolyte. More importantly, for the first time here we have used the redox-active electrolyte for the TMOs based asymmetric SCs device.

## Results

**Figure 1:**
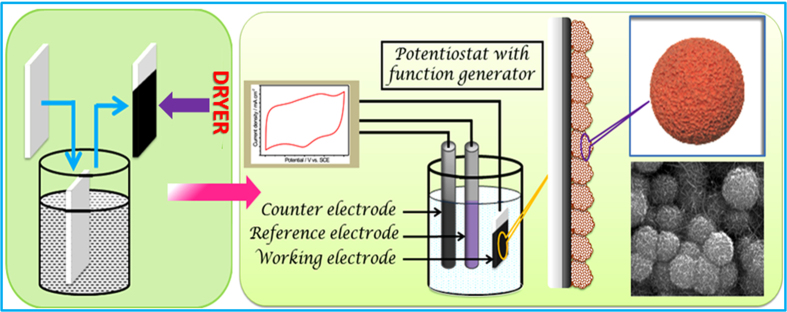
Schematic representation of steps involved in synthesis of MWCNTs/MnO_2_ nanocomposite thin film on stainless steel substrate where MWCNTs are coated by “dip and dry” method followed by electrodeposition of MnO_2_.

The synthesis of nanostructured MWCNTs/MnO_2_ electrode material by scalable path is the primary target of this work. The steps involved in the synthesis of MWCNTs/MnO_2_ hybrid thin films are presented in Fig. 1, while the actual experiment setup is shown in S.I. S1. The electrodeposition is very fascinating phenomenon; in which the deposition is occurring on the conducting substrate by passing current though the electrochemical cell. For SCs application, preparation of uniform and smooth porous nanostructures with nano-channels is very essential to improve the resultant energy storing capacity. In which, porous MnO_2_ spongy balls were developed on LBL deposited MWCNT network by single electrodeposition route. The highly porous MWCNT network allows for high mass loading of MnO_2_ as compared to the flat stainless steel (SS) substrates on the same projection area. The MWCNTs network also provides the high path for electrical conduction, which will help in improving the power density of active electrode material.

**Figure 2:**
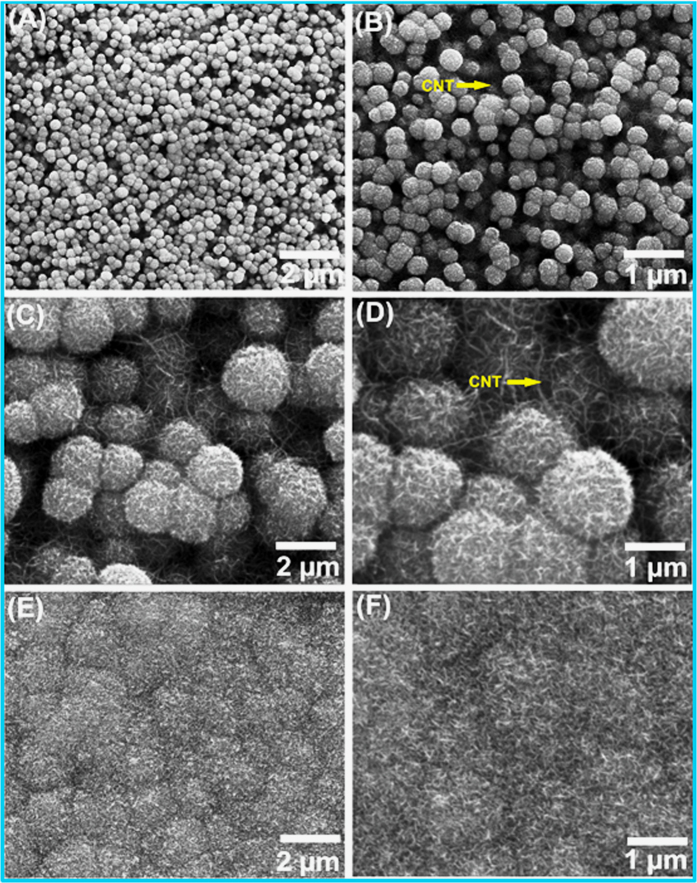
FESEM images of (**A**,**B**) CM10, (**C,D**) CM20 and (**E**,**F**) CM30 thin films at two different magnifications.

The visualization of surface morphology is very crucial for supercapacitor application, as the visualized surface gives idea regarding how effective electrochemical interactions occurs in between the active electrode material and the cations of the electrolyte^9^. The SEM images of LBL deposited MWCNT thin film on SS substrate is presented in S.I. Figure S3(A,B). The SEM images of MWCNT thin film shows porous needle like surface which is providing much more surface area for deposition MnO_2_ as compared to bare SS substrate. The SEM images of CM10, CM20 and CM30 thin films at two different magnifications shown in Fig. 2(A–F). The spongy balls like structure with ultrathin nanoflakes is clearly observed for CM10 thin film (see Fig. 2(A,B)). The average size of spongy balls for CM10 thin film is in the range of 200–300 nm. At higher magnification, it can be seen that the ultrathin nanoflakes with spongy balls of MnO_2_ provides the electronic transfer channel, which is expected to improve the conductivity and rate capacity of the obtained MnO_2_ nanostructure. The cluster of very fine nanoflakes of spongy balls generate abundant free space, that will provides easy path for electrolyte ions to make superior insertion and desertion in active electrode material. The increasing deposition cycles from 10 to 20, moderately change the surface morphology as shown in Fig. 2(C,D). As the deposition cycles of MnO_2_ increased to 20, the mass loading of MnO_2_ is effectively increased from 1.1 to 1.8 mg and that will increase the size of spongy balls (see Fig. 2D). In general, the high mass loading of MnO_2_ usually increases the electrode resistance which reduce the resultant electrochemical performance of active electrode. This is due to at a higher mass loading, MnO_2_ become densely packed and that will reduce the porosity as well as the specific surface area of the active electrode material. Further, increase in deposition cycles to 30 (see Fig. 1(E,F)), the extra thick layer of MnO_2_ is formed over MWCNTs. In which spongy ball like structure of MnO_2_ is collapsed and converted into compact dense like structure. This kind of surface reduces the porosity as well as the electroactive surface area by increasing the charge transfer resistance. Fine observation of SEM images from Fig. 2(A) to (F), the more porous surface is observed for CM10 thin film as compared to CM20 and CM30, which is more suitable for getting the higher electrochemical performance.

**Figure 3:**
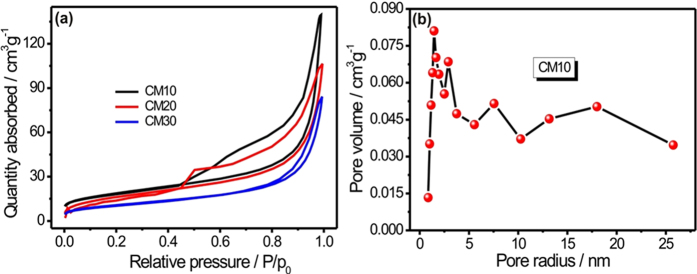
Nitrogen adsorption-desorption curves for CM10, CM20 and CM30 samples (**b**) The pore size distribution plot for CM10 sample.

To measure the actual specific surface area (SSA) and porosity of the MWCNTs/MnO_2_ thin-film, the nitrogen adsorption-desorption measurements are carried out. Figure 3(a) shows the nitrogen adsorption-desorption isotherms for all MWCNTs/MnO_2_ samples. The isotherms show type IV hysteresis loops, which is located in relatively high pressure region (0.45–1.0 P/p_0_), indicating the mesoporous nature of all sample. In general, the type IV hysteresis loop is mostly occurring due to the aggregated nanosheets like structure. Further, the SSA was calculated using the Brunauer-Emmett-Teller (BET) method. The BET surface area for CM10, CM20 and CM30 samples is 94, 63, 38 m^2^ g^−1^, respectively. The calculated SSA for the CM10 sample is about 94 m^2^g^−1^, which is much higher than previously reported MnO_2_ samples^20^,^21^. The results of BET analysis are well analogous with the SEM study. As the size of the spongy ball increased from the CM10 to CM30 thin films, the effective SSA is decreased from 94 to 38 m^2^ g^−1^ and as far as deals with the SC application higher SSA is desired to get excellent electrochemical performance. Figure 3(b) shows the BJH pore size distribution plot for CM10 sample. The most of the pores are observed within the range of 2 to 20 nm and centred at 2.06, 2.94 and 7.55 nm indicating the mesoporous nature of the CM10 sample. This mesoporous structure could provide the easy access for electrolyte ions as well as short diffusion path for intercalation and deintercalation^19^.

**Figure 4:**
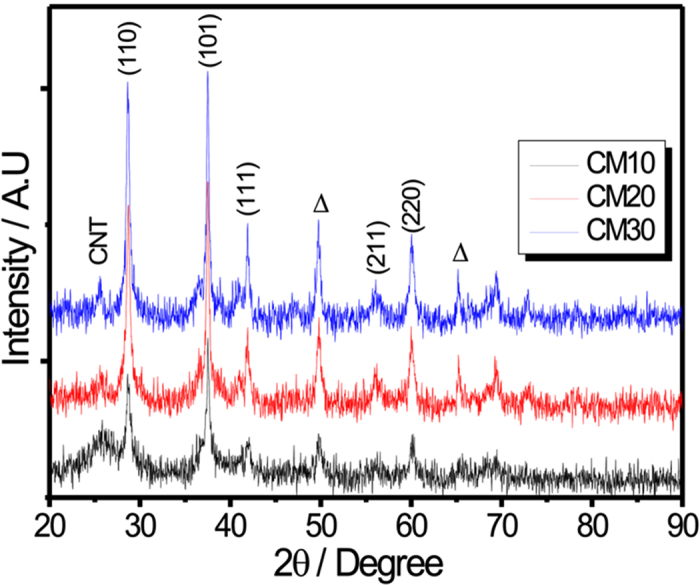
XRD patterns for CM10, CM20 and CM30 thin films on stainless steel substrate.

**Figure 5:**
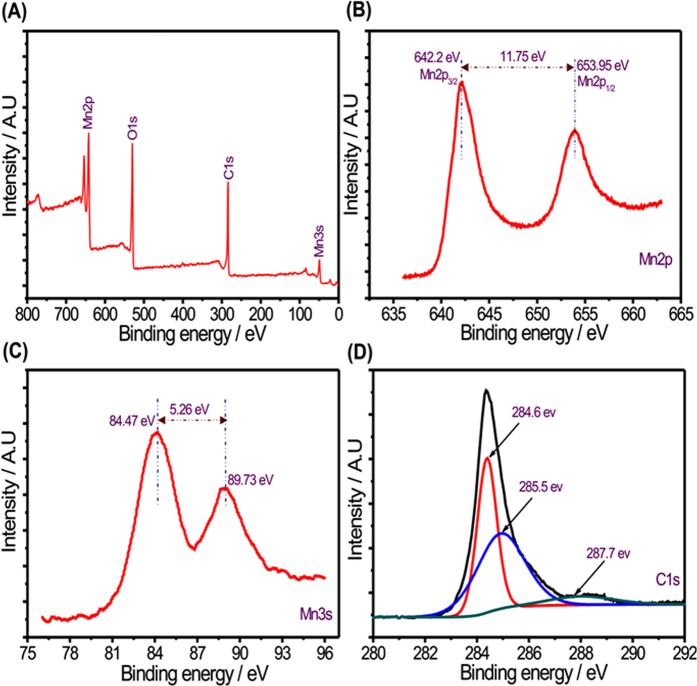
(**A**) Surface scanning XPS full spectrum for CM10 thin film, (**B**) Mn2p, (C) Mn3s and (**D**) C1s core level XPS spectra of the CM10 thin film.

The crystal structures of all MWCNTs/MnO_2_ thin films were characterized by XRD as shown in Fig. 4. It is clearly observed that all the diffraction peaks can be indexed to tetragonal MnO_2_ phase (JCPDS no. 34–1266), which is consistent with literature. Further, the little increment in peak intensity is observed from CM10 to CM30 thin film, this is due to the mass loading of MnO_2_ increases from the CM10 to CM30 thin films. For MWCNT, the broad peak is observed at 2θ of 25.5° having very low intensity. To further detect the composition and surface chemical states of as-prepared MWCNTs/MnO_2_ thin-film, the XPS analysis of CM10 thin film was performed and displayed in Fig. 5(A–D). The broad XPS spectra of CM10 thin film showing only three kind of elements (Mn, O and C) suggesting the best purity of prepared composite thin film (Fig. 5(A)). The narrow scan XPS spectrum of Mn2p exhibits multiple splitting with two main peaks at binding energies of 642.2 and 635.95 eV which can be assigned to Mn2p_3/2_ and Mn2p_1/2_ of Mn^4+^ in MnO_2_, respectively (Fig. 5(B)). In general, the oxidation state of Mn in manganese oxide is determined by considering the energy difference in Mn3s doublet. The narrow scan XPS spectrum of Mn3s is shown in Fig. 5(C). The energy difference between Mn3s doublet is 4.95 eV, suggesting the Mn has IV oxidation state in prepared manganese oxide. Figure 5(D) showing the narrow scan spectrum of C1s, the characteristic peaks are well match for MWCNTs^22^,^23^.

**Figure 6:**
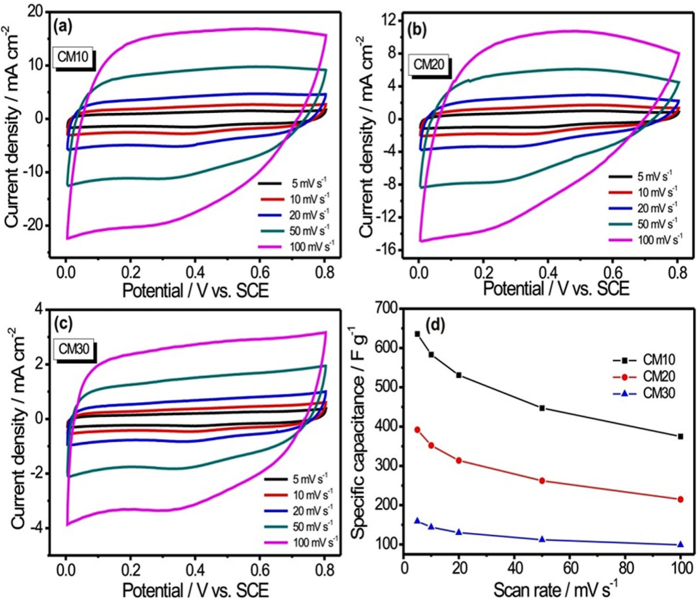
The CV curves for (**a**) CM10, (**b**) CM20 and (**c**) CM30 thin films at various scan rates from 5–100 mV s^−1^ and (**d**) the plots of specific capacitance with scan rate for all MWCNTs/MnO_2_ nanocomposite thin films.

To decide the best electrode for supercapacitor application, the electrochemical performance of all three MWCNTs/MnO_2_ thin films are evaluated in a three-electrode configuration with 1 M Na_2_SO_4_ electrolyte. The CV measurements were carried out at various scan rates ranging from 5–100 mV s^−1^. Figure 6(a–c) shows a set of rate-dependent CV curves for all three MWCNTs/MnO_2_ thin films. It can be seen that all the CV curves have nearly rectangular shape, indicating superior supercapacitor behaviour even at high scan rate of 100 mV s^−1^. Enhancement in current response with scan rate is observed for all three systems, which suggests the effective utilization active electrode material by electrolyte ions. The specific capacitances of all three MWCNTs/MnO_2_ thin films were calculated on the basis of their CV curves, and plotted as a function of the scan rate in Fig. 6(d). To achieve the highest specific capacitance for MnO_2_, the mass loading of MnO_2_ needs to be infinitely small to minimize the bulk resistance and the scan rate needs to be very low to improve electrons and ions transport in active electrode material. In present study, when the mass loading of MnO_2_ on the MWCNTs network was increased from 1.1 to 2.6 mg cm^−2^, the value of specific capacitance drastically decreased from the 636 to 158 F g^−1^ at scan rate of 5 mV s^−1^ (see S. I. S5 for calculations) The value of specific capacitance dropped from CM10 to CM30 thin film. This is due to as the mass loading of MnO_2_ increased from CM10 to CM30 thin film, the porosity, specific surface area and the conductivity of active electrode material decreased and that will directly affect the resultant electrochemical performance of active electrode.

**Figure 7:**
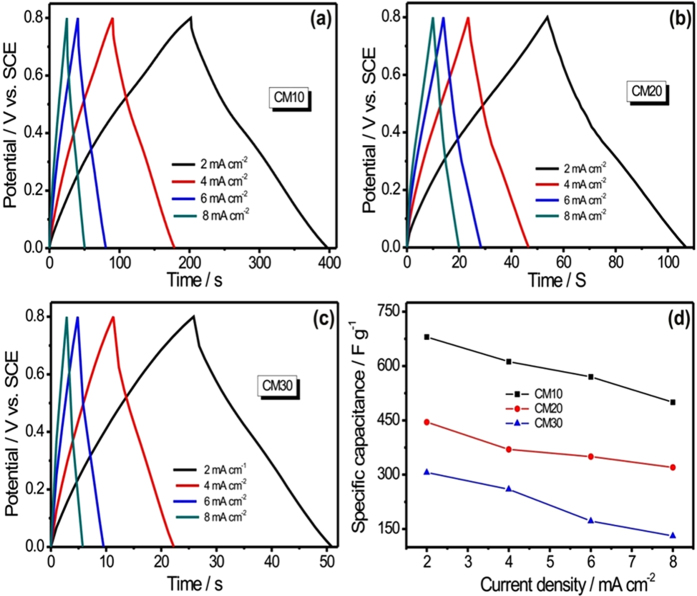
The charge discharge curves for (**a**) CM10, (**b**) CM20 and (**c**) CM30 thin film at various current densities ranging from 2–8 mA cm^−2^ and (**d**) the plots of specific capacitance with current density for all MWCNTs/MnO_2_ nanocomposite thin films.

The capacitive behaviour of all three MWCNTs/MnO_2_ thin films were further studied by measuring the GCD at various current densities ranging from 2 to 8 mA cm^−2^, shown in Fig. 7(a–c). The GCD curves for all three MnO_2_@MWCNTs thin films shows good linear potential-time profiles, demonstrating a good capacitive performance. The plot of specific capacitance as a function of current density, as determined from the GCD curves, is shown in Fig. 7(d). The CM10 thin film shows high specific capacitance of 680 F g^−1^ than the CM20 (444 F g^−1^) and CM30 (305 F g^−1^) thin films. The obtained value of specific capacitance for CM10 thin film is much higher as compared to the previous reports (see S. I. S6). Further, it is found that the specific capacitance decreases with the increase in current density. The CM10 thin film shows the excellent rate capability by retaining the 75% of initial specific capacitance even at high current density of 8 mA cm^−2^. While the CM20 and CM30 thin films shows moderate rate capability by retaining the 67 and 44% of initial specific capacitance after charging with current density of 8 mA cm^−2^. The low capacitance is observed at high current density can be attributed to the decrease in the utilization efficiency of the active electrode material by electrolyte ions.

**Figure 8:**
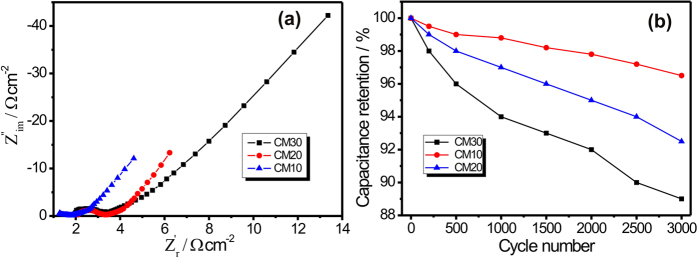
(**a**) Nyquist plots for CM10, CM20 and CM30 thin films at identical conditions and (**b**) plots of capacity retention with cycle number for all MWCNTs/MnO_2_ thin films.

The EIS is measured by applying the constant bias potential of 10 mV within frequency range of 100 mHz to 100 kHz. The obtained Nyquist plots for all three MWCNTs/MnO_2_ thin films are shown in Fig. 8(a). The Nyquist plot clearly shows the effects of MnO_2_ loading on the small semicircle, Warburg diffusion line and capacitive line. First important parameter obtained from the Nyquist plot is the equivalent series resistance (ESR). In general, the first intercept of the Nyquist plot to the real axis in the high frequency range offers the ESR. The ESR includes the internal resistances of the active material, bulk resistance of electrolyte, and the interfacial contact resistance between electrolyte and electrode. Second, the charge transfer resistance (Rct) can be calculated from the diameter of semicircle in the high frequency region, which arises because of diffusion of electrolyte ions/electrons in the active electrode material. The Warburg impedance describes the diffusion of electrolyte species as well as the non-uniform nature of active electrode material, can be calculated from the slope of the EIS curve. In the lower frequency region, the vertical line parallel to the imaginary axis indicates an ideal capacitive behaviour of the active electrode material. In present case, the obtained ESR values for CM10, CM20 and CM30 thin film are 1.27, 1.84 and 2.16 Ω cm^−2^, respectively. The slightly lower value of ESR for CM10 thin film is may be due to the strong bounding between the current collector to MWCNT and MWCNT to spongy balls of MnO_2_. Further, a relatively very small semicircle in the high frequency region is observed for CM10 thin film than the CM20 and CM30 thin film. The calculated values Rct for CM10, CM20 and CM30 thin films are 0.53, 1.23, 1.60 Ω cm^−2^, respectively. The amplitude of Rct is higher for CM20 and CM30 thin film as compared to the CM10 thin film suggesting the slower electron transfer for CM20 and CM30 thin films. The Nyquist plot for bare SS electrode in 1 M Na_2_SO_4_ electrolyte is shown in S I S10 and which shows very high resistance as compared to the prepared MWCNTs/MnO2 thin films. The electrochemical cycling stability is another substantial issue that finds out the practical application of SCs. For real SCs application, the excellent cycling stability for active electrode material is essential. The stability test for all three electrodes MWCNTs/MnO_2_ thin films are carried out by measuring the CV for 3000 cycles at constant scan rate of 100 mV s^−1^. Figure 8(b) compares the cycling stability of all three electrodes over 3000 CV cycles and shows 96, 92.3 and 88.7% capacity retention for CM10, CM20 and CM30 thin films, respectively. All three systems demonstrate the better cycling stability as compared to previous report (see S. I. 6). The slightly better electrochemical stability is observed for the CM10 thin film as compared to CM20 and CM30 thin film. This suggests the strong bounding of spongy balls (for CM10 thin film) with MWCNTs network and that will help for stabilizing the MnO_2_ nanoparticles mechanically on MWCNTs network.

**Figure 9:**
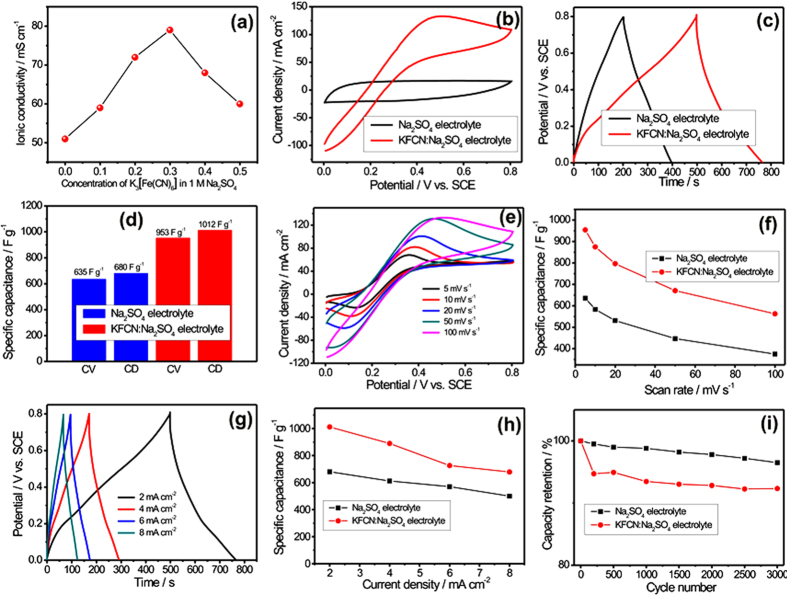
(**a**) The plot of ionic conductivity with concentration of K_3_[Fe(CN)_6_] in 1 M Na_2_SO_4_ electrolyte, (**b**) CV curves for CM10 thin film in Na_2_SO_4_ and KFCN:Na_2_SO_4_ electrolyte at constant scan rate of 100 mV s^−1^, (**c**) the charge-discharge curves for CM10 thin film in Na_2_SO_4_ and KFCN:Na_2_SO_4_ electrolyte at constant current density of 2 mA cm^−2^, (**d**) comparative bare diagram showing the specific capacitance for CM10 thin film in Na_2_SO_4_ and KFCN:Na_2_SO_4_ electrolyte, (**e**) CV curves for CM10 thin film at various scan rates ranging from 5–100 mV s^−1^ in redox electrolyte, (**f**) plot of specific capacitance with scan rate for CM10 thin film in Na_2_SO_4_ and KFCN:Na_2_SO_4_ electrolyte, (**g**) the charge discharge curves for CM10 thin film at various current densities ranging from 2–8 mA cm^−2^, (**h**)) plot of specific capacitance with current densities for CM10 thin film in Na_2_SO_4_ and KFCN:Na_2_SO_4_ electrolyte, (**i**) plot of capacity retention with 3000 CV cycles for CM10 thin film in Na_2_SO_4_ and KFCN:Na_2_SO_4_ electrolyte.

Till time to improve the electrochemical perormance of the SC different active electrode materials are explored. The different nanostructures of different active electrode materials have been explored to get higher electrochemical performance^22^,^23^. On the same time, the electrolytes are supposed to be an inert component of SC but it is not literal information. The electrolytes are not a least component of SC, which can effectively moderate the resultant electrochemical performance^24^. Therefore, it is essential to focus on the electrolyte system so as to improve the electrochemical performance of the SC. In recent years, the different redox active species are doped in the parent electrolyte system to enhance electrochemical features of electrolyte^22^,^23^,^25^,^26^,^27^. The electrolytes with redox active species are capable to undergoing redox reactions on the surface of active electrode material. Previously, redox electrolyte majorly used for the carbon and conducting polymer based electrodes^22^,^23^,^28^. Inspired by these work, for the first time we are reporting the successful example of the redox additive aqueous electrolyte for the metal oxide based electrode (MWCNTs/MnO_2_ thin film). Simply, the conventional aqueous 1 M Na_2_SO_4_ electrolyte is doped with redox active species (K_3_[Fe(CN)_6_]). To achive higher ionic conductivity for electrolyte and better electrochemical perfromance for MWCNTs/MnO_2_ thin film, the different amount of K_3_[Fe(CN)_6_] (from 0.1 to 0.4 M) were added in 1 M Na_2_SO_4_ electrolyte and tested for ionic conductivity measurmant. Figure 9(a) shows the plot of ionic conductivity versus the concentration of K_3_[Fe(CN)_6_] in 1 M Na_2_SO_4_ electrolyte. It is observed that the ionic conductivity value increses with the corresponding K_3_[Fe(CN)_6_] concentration up to 0.3 M K_3_[Fe(CN)_6_]. However, when the concentration of K_3_[Fe(CN)_6_] reaches to 0.4 M in 1 M Na_2_SO_4_, the value of ionic conductivity effectively decreased. This is because of at higher concentration of K_3_[Fe(CN)_6_] in 1 M Na_2_SO_4_ leads to the aggregation of free ions and the crystallization of K_3_[Fe(CN)_6_] in Na_2_SO_4_ system. Therefore, to measure the electrochemical performance of CM10 thin film, we have selected the electrolyte system which consists the 0.3 M K_3_[Fe(CN)_6_] in 1 M Na_2_SO_4_. Further, the conventional Na_2_SO_4_ and the redox additive aqueous {0.3 M K_3_[Fe(CN)_6_] + 1 M Na_2_SO_4_} electrolytes abbreviated as the Na_2_SO_4_ and KFCN:Na_2_SO_4_ electrolyte, respectively.

**Figure 10:**
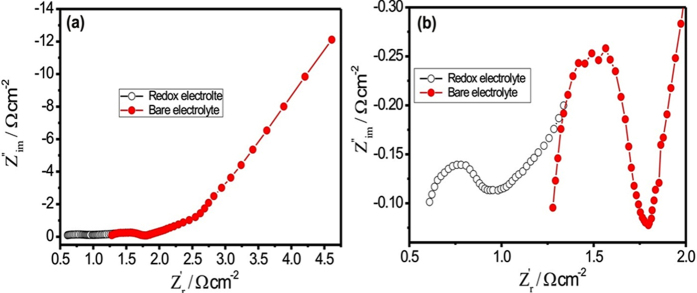
(**a**) Nyquist plot for CM10 thin film in Na_2_SO_4_ and KFCN:Na_2_SO_4_ electrolyte at identical conditions and (**b**) the enlarge view of Nyquist plot.

The CV curves of CM10 thin film in Na_2_SO_4_ and KFCN:Na_2_SO_4_ electrolytes at constant scan rate of 100 mV s^−1^ is presented in Fig. 9(b). The CV curve for KFCN:Na_2_SO_4_ electrolytes shows very high amplitude of current as compared to the Na_2_SO_4_ electrolyte. Additionally, the CV curve for redox electrolyte deviates from the idea rectangular shape and shows the redox peaks which result in higher energy storing capacity. In KFCN:Na_2_SO_4_ electrolyte, [Fe(CN)_6_]^3−^/[Fe(CN)_6_]^4−^ redox couple formed and therefore additional pseudocapacitance could be easily generated from the redox reaction between [Fe(CN)_6_]^3−^ and [Fe(CN)_6_]^4−^, which helps in improving the energy storing capacity of the CM10 thin film. The GCD measurements are also carried out in both electrolyte systems at constant current density of 2 mA cm^−2^, shown in Fig. 9(c). As observed in GCD curves, the CM10 thin film shows better electroactivity in KFCN:Na_2_SO_4_ electrolyte, as it take more time for charge and discharge than the bare electrolyte, which is analogous to the CV curves. Further, the specific capacitance for CM10 thin film is calculated for both electrolyte system using the CV and GCD curves at identical scan rate and current density. Figure 9(d) shows the plot of specific capacitance for CM10 thin film in both electrolyte systems. As observed from figure, the CM10 thin film shows approximately 1.5-fold higher specific capacitance in redox electrolyte as compared to the conventional electrolyte. The specific capacitance for CM10 thin film is enhanced from 635 to 953 F g^−1^ (From CV) and 680 to 1012 F g^−1^ (From GCD). The addition of redox additive in conventional electrolyte provides extra electrons for electrochemical reactions; it improves the ionic conductivity of electrolyte and also moderates the compatibility of electrolyte to the active electrode material. In addition to this, the nano porous surface morphology, higher specific surface area and appropriate mass loading of MnO_2_ on MWCNTs provide not only high electroactive sites for electrolyte ions but also provide higher conductivity for electron transportation. That why we obtained very high specific capacitance for CM10 thin film in redox electrolyte than the previous reports (see S I S6). In next to calculate the rate capability of CM10 thin film in KFCN:Na_2_SO_4_ electrolyte, CV and GCD measurements are carried out at various scan rates. Figure 9(e,g) shows the CV and GCD curves of CM10 thin film in KFCN:Na_2_SO_4_ electrolyte, respectively. By using these CV and GCD curves, the specific capacitance is calculated for both electrolyte system and plotted in Fig. 9(f,h) as a function of scan rate and current density. Figure 9(f,h) clearly shows the KFCN:Na_2_SO_4_ electrolyte is the best for CM10 thin film, as it gives higher specific capacitance than the Na_2_SO_4_ electrolyte. The maximum obtained specific capacitance for CM10 thin film is the 1012 F g^−1^ and which is slightly less than the theoretical specific capacitance (1370 F g^−1^) of MnO_2_. More importantly, the obtained value of specific capacitance for CM10 thin film in KFCN:Na_2_SO_4_ electrolyte is much higher than the previous report (see S. I. S6) and that proves the potential of the present work for developing the higher energy storage devices^29^,^30^,^31^,^32^,^33^,^34^. Figure 9(i) compares the cycling stability of the CM10 thin film in Na_2_SO_4_ and KFCN:Na_2_SO_4_ electrolyte. To measure the cycling stability CV cycling repeated for 3000 times at constant scan rate of 100 mV s^−1^. It is noticed that the cycling stability of CM10 thin film in KFCN:Na_2_SO_4_ electrolyte slightly lesser as compared to the Na_2_SO_4_ electrolyte. The redox electrolyte performs the intensive redox reactions with active electrode material, which moderately affect/loss the electroactive sites and also it improves the rate of dissolution of active electrode material in electrolyte. In present case, the obtained maximum capacity retention for CM10 thin film over 3000 CV cycles in KFCN:Na_2_SO_4_ electrolyte is 92.33%. The high cycling stability for CM10 thin film even in the redox electrolyte is mainly contributed by synergetic effect of MWCNTs and MnO_2_. The MnO_2_ spongy balls tightly bounded with MWCNTs network that does not allow easy dissolution of active electrode material in electrolyte, which keep the high cycling stability even after 3000 CV cycles. The Nyquist plot for CM10 thin film in Na_2_SO_4_ and KFCN:Na_2_SO_4_ electrolyte measured at identical conditions and plotted in Fig. 10(a). The enlarge view of Nyquist plot is shown in Fig. 10(b), which clearly shows the effect of redox electrolyte on the electrochemical performance of CM10 thin film. The CM10 thin film demonstrates very low Rs and Rct in KFCN:Na_2_SO_4_ electrolyte as compared to the Na_2_SO_4_ electrolyte. Simply this is due to the higher ionic conductivity of the redox electrolyte allows fast, easy and large electrons for electrochemical reactions.

**Figure 11:**
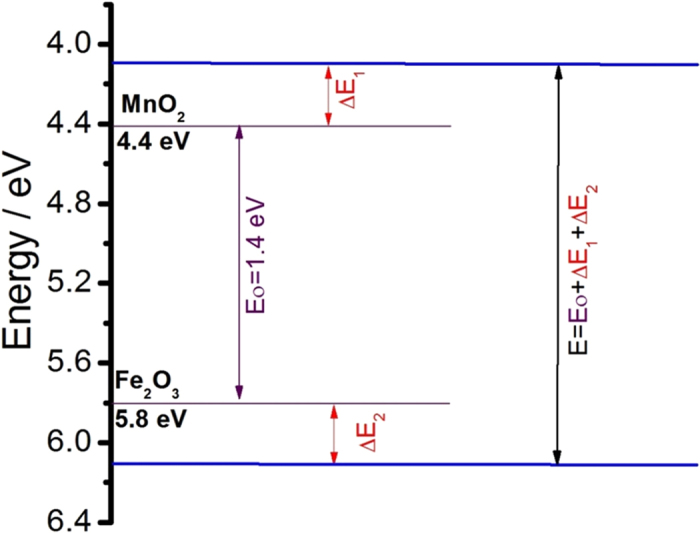
Schematic presentation of work function for MnO_2_ and Fe_2_O_3_ with the relationship between potential window and the shift of work function during charging of two electrodes.

To inferring any conclusion from electrochemical results of the 3 electrodes system is not so much appropriate, therefor we fabricated the asymmetric SCs (2 electrodes system) using the MWCNTs/MnO_2_ and Fe_2_O_3_ as a cathode and anode (after balancing the charges of both electrode), respectively with the KFCN:Na_2_SO_4_ electrolyte. Simply we prepared the Fe_2_O_3_ thin film using sucessive ionic layer adsorption and reaction (SILAR) method as reported previouly by our group^15^. The reason behind the selecting Fe_2_O_3_ as an anode electrode is due to its complementry operating potential window in neutral electrolyte compared to the MnO_2_^15^. Another reason for selecting the Fe_2_O_3_ as a negative electrode due to its higher work function as compared to the MnO_2_. In general, the operating potential window of the metal oxides based asymmetric SCs is calculated using the following equetion^28^,


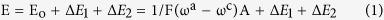


where, E is the resultant operating potential window for asymmetric SCs, ω^a^ and ω^c^ are the work function for the anode and cathode material, ΔE_1_ and ΔE_2_ are the surfece dipole potential for cathod and anode, respectively. As the symmetric SCs based on the identical material (ω^a^ = ω^c^), the resultant potantial limit is decided by the electrolyte. On the other hand, the asymmeric SCs is based on the different electrode material (ω^a^ ≠ ω^c^). Therefore, there is possibility for getting higher operating potential window. Figure 11 shows the work function plot for MnO_2_ and Fe_2_O_3_^28^. From equetion 1, the calculated operating potential window for the asymmetric SCs is 1.4 V. Further, the chemisorption of electrolyte ions (cations, anions, redox species, hydroxide ions) on the surface of the active electrode modifies the work function and that will again extend the operating potential window of the asymmetric SCs device (see Fig. 11).

**Figure 12:**
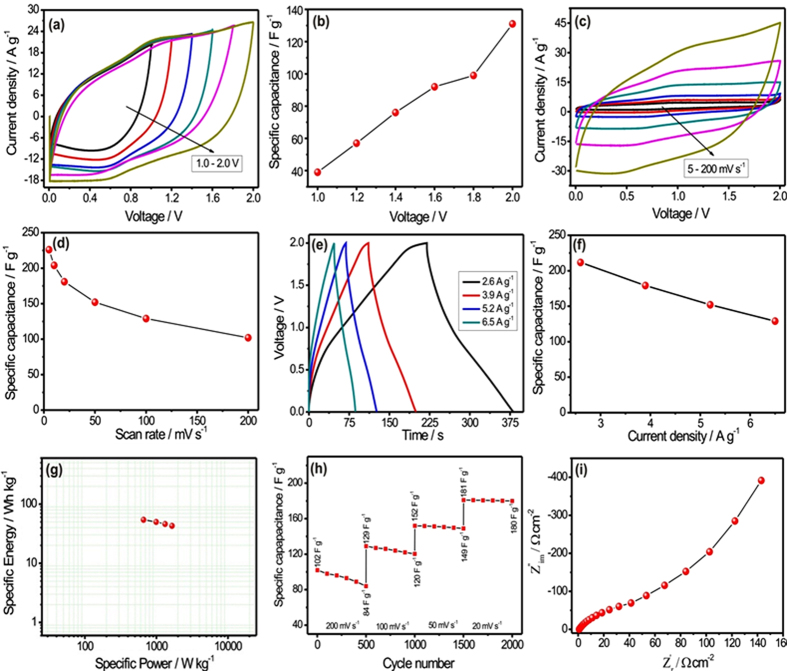
(**a**) CV curves for MWCNTs/MnO_2_//Fe_2_O_3_ asymmetric SCs at different operating potential windows in KFCN:Na_2_SO_4_ electrolyte at constant scan rate of 100 mV s^−1^, (**b**) the plot of specific capacitance with different operating potential window for MWCNTs/MnO_2_//Fe_2_O_3_ asymmetric SCs, (**c**) CV curves at various scan rates ranging from 5 to 200 mV s^−1^ for MWCNTs/MnO_2_//Fe_2_O_3_ asymmetric SCs, (**d**) plot of specific capacitance with scan rate for MWCNTs/MnO_2_//Fe_2_O_3_ asymmetric SCs, (**e**) the charge-discharge curves at various current densities for MWCNTs/MnO_2_//Fe_2_O_3_ asymmetric SCs, (**f**) plot of specific capacitance with current densities for MWCNTs/MnO_2_//Fe_2_O_3_ asymmetric SCs (**g**) Ragone plot for MWCNTs/MnO_2_//Fe_2_O_3_ asymmetric SCs, (**h**) plot of capacity retention with CV cycles for MWCNTs/MnO_2_//Fe_2_O_3_ asymmetric SCs at different scan rate and (**i**) Nyquist plot for asymmetric SCs.

The first important task for SCs device is the optimisation of appropriate opertaing potential window in order to maintain the electractivity of active electrodes and electrolyte. Therefore, CV measurment is carried out at different operating potential window rainging from the 1.0 to 2.0 V shown in Fig. 12(a). The CV curves show the better capacitive features by maintaining the rectangular shape even at higher potential window of 2.0 V. It is noted that the stable electrochemical window of the asymmetric SCs can be extended to 2.0 V. The increasing operating potential window moderately enhances the specific capacitance of the asymmetric SCs device shown in Fig. 12(b). Further, the CV measurement is carried out at different scan rates from 5 to 200 mV s^−1^ (see Fig. 12(c)). The maximum specific capacitance of 226 F g^−1^ is obtained at lower scan rate of 5 mV s^−1^, while it is decreased to 102 F g^−1^ at higher scan rate of 200 mV s^−1^ by showing moderate rate capability (see Fig. 12(d)).

Figure 12(e) demonstrates the GCD curves at different current densities of 2.6, 3.9, 5.2 and 6.5 A g^−1^ within operating potential window of 0 to 2 V. The GCD curves for different working voltage windows are shown in S. I. S8. Meanwhile, the GCD curves show the nonlinear behavior with small voltage drops at the initial region of the discharge curve. From GCD curves, the maximum specific capacitance of 211 F g^−1^ is obtained at lower current density of 2.6 A g^−1^. Furthermore, to study the efficiency of fabricated asymmetric SCs device, specific energy and specific power are calculated and compared with previous reports (see S. I. S9). Figure 12(f) shows the Ragone plot at different current densities. The asymmetric SCs device achieved maximum energy density of 54.39 Wh kg^−1^ at power density of 667 W kg^−1^. The calculated energy density is acceptable and much higher as compared to previous asymmetric SCs devices^35^,^36^,^37^,^38^,^39^,^40^,^41^,^42^. Cycling stability of the asymmetric SCs device is measured at different scan rate from 200 mV s^−1^ to 20 mV s^−1^ to get idea regarding the reliability of the device at different scan rate. Figure 12(h) depicts the graph of specific capacitance with cycle number. Firstly, the asymmetric device is operated at higher scan rate of 200 mV s^−1^ for 500 CV cycles and for that moderate capacity retention (83%) is observed (see S. I. S10). Further, as scan rate diminishes from 200 to 20 mV s^−1^, the improvement in capacity retention is observed (from 83 to 99%). The cycling stability data confirms that the asymmetric device is stable at higher as well as lower scan rate. Figure 12(i) shows the Nyquist plot for the asymmetric SCs device. The asymmetric SCs device shows very low ESR (0.20 Ω) in high frequency region, which suggests low series resistance of the SC, benefiting from the excellent conductivity of electrolyte and electrodes.

## Discussion

**Figure 13:**
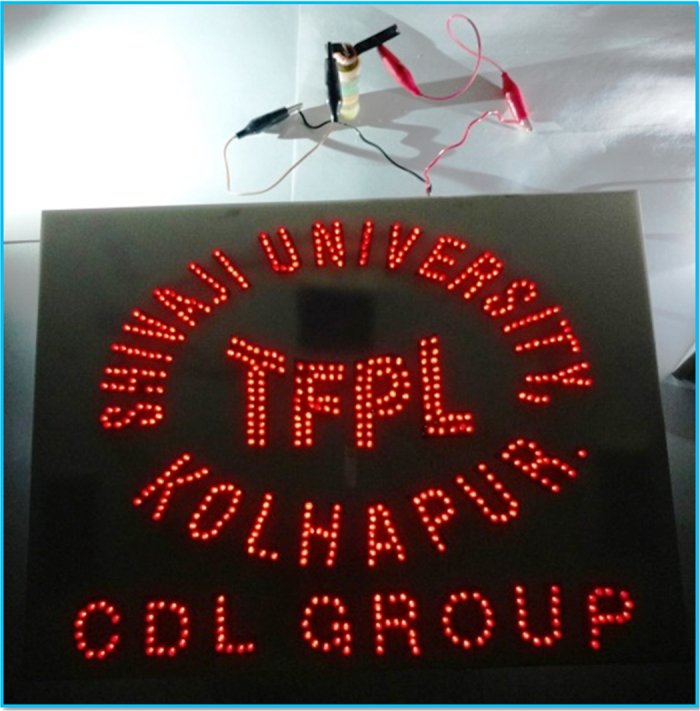
Digital photographs of actual demonstration of MWCNTs/MnO_2_//Fe_2_O_3_ asymmetric device by lighting the panel of 567 LEDs with the text of “SHIVAJI UNIVERSITY, KOLHAPUR, TFPL, CDL GROUP”.

As described in the above, the excellent electrochemical performance for the fabricated asymmetric SCs devices (MWCNTs/MnO_2_//Fe_2_O_3_) can be reasonably attributed to the synergistic contribution of both electrode materials (MWCNTs/MnO_2_ and Fe_2_O_3_) and the redox electrolyte. The excellent electrochemical results are observed for the asymmetric SC device due to the interactive combination of nanostructured MWCNTs/MnO_2_, Fe_2_O_3_ and redox-active electrolyte. The enhanced capacitive performances for asymmetric SCs device would result from 1) the advantage of both charge storage mechanism, MWCNTs via EDLC while MnO_2_ stores charges by psedocapacitive mechanism, 2) The conducting MWCNTs network not only provides the high path for electric conduction but also it can provide the porous surface for ion adsorption and MnO_2_ hosting, 3) the spongy balls of MnO_2_ provides much more electroactive sites for electrochemical reactions 4) the nanostructured Fe_2_O_3_ thin film can store the electrical charges by psedocapacitive mechanism, that will again help for enhancing the energy density of the asymmetric SCs device 5) last but not least, the use of an inexpensive and redox capable KFCN:Na_2_SO_4_ electrolyte brought enormous capacitive contributions for both thin film through the reversible faradic reactions. Additionally, the results of asymmetric SCs device discussed in present manuscript are superior as compared to earlier reported in terms of 1) use of very simple bottom-up approach for thin film synthesis 2) use of cost effective and flexible SS substrate with cheap electrode materials and 3) very easy route for assembling the asymmetric SCs device. In addition, just to show the work capability of the assembled asymmetric SCs device, we demonstrated that the panel of 567 red light emitting diodes (LEDs) texted in “SHIVAJI UNIVERSITY, KOLHAPUR, TFPL, CDL GROUP” could be powered for 1 min by single MWCNTs/MnO_2_//Fe_2_O_3_ asymmetric SCs device after charging for 20 s with 2.5 V (see Fig. 13). More importantly, the glowing intensity of LEDs suggesting the high power capability of the MWCNTs/MnO_2_//Fe_2_O_3_ asymmetric SCs device, while the extended glowing time period of LEDs suggesting the higher energy storing capacity of the MWCNTs/MnO_2_//Fe_2_O_3_ asymmetric SCs device. Therefore, the approach discussed in present study will offer a valuable and promising tool for producing highly energetic energy storage devices for advance electronics.

In summary, a new efficient approach–hybrid electrode with redox-active electrolyte has been developed to assemble the highly efficient MWCNTs/MnO_2_//Fe_2_O_3_ asymmetric SCs devices. The unique MWCNTs/MnO_2_ architectured hybrid film showed excellent electrochemical features with maximum specific capacitance of 1012 F g^−1^ at 2 mA cm^−2^ current density in redox-active electrolyte, which is 1.5-fold higher than that of conventional electrolyte. Specifically, the developed MWCNTs/MnO_2_//Fe_2_O_3_ asymmetric SCs device shows high specific capacitance (226 F g^−1^), high specific energy (54.39 Wh kg^−1^), and good cycling stability (over 2000 cycles) at an operation potential window of 2.0 V in redox-active electrolyte. Strikingly, actual practical demonstration shows lightning of 567 red LEDs suggesting “ready-to sell” product for industries. Lastly, we believe that the proposed hybridization approach for the electrode and electrolyte open up the advance way for developing the energy storage devices having high energy storing capacity.

## Methods

### Synthesis of MWCNTs/MnO_2_ thin films

The multi-walled carbon nanotubes (MWCNT) prepared by a conventional CVD method were purchased from Sigma Aldrich (95%, outer diameter 10–15 nm, length 1–1 mm). As-purchased MWCNTs were refluxed in concentrated H_2_SO_4_/HNO_3_ (3:1) at 353 K, as reported previously^18^, in order to remove the amorphous carbon content and to attach the carboxylic and/or hydroxyl groups on the walls. Further, the functionalized MWCNTs were stirred for 12 h in DDW and then subjected to centrifuge at 2000 rpm. In the next step, the functionalized MWCNTs were washed with DDW until the pH 7 and dried in vacuum at temperature 343 K overnight. Later, 0.01 g of functionalized MWCNTs was sonicated in 50 ml DDW for 3 h to form stable dispersion which was used as precursor solution for the synthesis of MWCNT thin films. Well cleaned SS substrate was dipped in MWCNTs solution for 30 s to adsorb the CNTs on SS surface owing to electrostatic attractive force between the SS substrate and CNTs in the solution^19^. Further, the SS substrate with MWCNTs was dried using air-drier which completes single deposition cycle. Such a 30 cycles were performed to get well adherent MWCNTs film on the SS substrate.

Later, porous MnO_2_ spongy balls were deposited on previously prepared MWCNTs by triple-electrode electrodeposition method. In which, saturated calomel electrode (SCE) was used as reference electrode, graphite as counter electrode and MWCNTs thin film used as working electrode. Briefly, 0.1 M MnSO_4_ and 0.1 KOH were dissolved in 50 ml double distilled water (DDW) at pH of 5.5 to avoid the production of Mn(OH)_2_ in the electrolyte solution. The porous MnO_2_ spongy balls were deposited on MWCNTs within potential limit of +0.4 to +1.4 V/SCE at scan rate of 50 mV/s. Simply, the deposition of MnO_2_ was carried out for different cyclic voltammetry (CV) cycles (10, 20 and 30 cycles) to optimize the mass loading of MnO_2_ on MWCNT network. The schematic of MWCNTs/MnO_2_ thin film synthesis is shown in Fig. 1, while the actual experiment setup is shown in supporting Fig. 1. The thin films with 10, 20 and 30 MnO_2_ CV cycles on MWCNT network were announced by CM10, CM20 and CM30, respectively. The mass of MnO_2_ in CM10, CM20 and CM30 thin films are 1.1, 1.8 and 2.6 mg cm^−2^ measured using ultrahigh sensitive weigh balance.

### Synthesis of Fe_2_O_3_ thin film

In present work, the successive ionic adsorption and reaction (SILAR) method was used to prepare Fe_2_O_3_ thin films on the SS substrate. For the preparation of Fe_2_O_3_ thin films, 0.05 M FeCl_3_ (cationic precursor) and 0.1 M NaOH (anionic precursor) was used in the 50 ml DDW. In the first step, the SS substrates were immersed in the FeCl_3_ solution for 10 s to adsorb Fe ions on the substrate surface. Then the substrates with Fe ions were rinsed in 50 ml DDW to remove loosely bound cations. Further, the substrate was inserted in the NaOH solution for 10 s, where the chemical reaction occurs in between pre-adsorbed Fe ions and OH ions and that will form the single layer of Fe_2_O_3_. Finally, the substrates were rinsed in 50 ml DDW to remove unreacted or excess species. This process was repeated for 100 SILAR cycles to obtain the desired thickness for Fe_2_O_3_ thin film.

### Characterization techniques

MWCNTs/MnO_2_ thin films were characterized by X-ray diffraction (XRD) analysis for structural study using a Bruker AXS D8 Advance Model with copper radiation (Kα of λ = 1.54°A). The surface morphology of the prepared thin films was investigated though field‒emission scanning electron microscopy (FE-SEM, JEOL JSM 6390). The N_2_ adsorption-desorption measurement was carried out to calculate the specific surface area and pore volume of MWCNTs/MnO_2_ thin films using ASAP-2010 surface area analyser. The electrochemical measurements were carried out using an Automatic Battery Cycler (WBCS3000) with a three electrode system containing MWCNTs/MnO_2_ thin film as a working electrode, platinum as a counter electrode and SCE as a reference electrode in 1 M Na_2_SO_4_ solution as an electrolyte. The impedance measurements were carried out using electrochemical impedance workstation (ZIVE SP5) within frequency range of 100 kHz to 100 mHz. The mass loading of MnO_2_ on current collector is measured by high precious micro-balance.

## Additional Information

**How to cite this article**: Chodankar, N. R. *et al*. An innovative concept of use of redox-active electrolyte in asymmetric capacitor based on MWCNTs/MnO_2_ and Fe_2_O_3_ thin films. *Sci. Rep.* **6**, 39205; doi: 10.1038/srep39205 (2016).

**Publisher's note:** Springer Nature remains neutral with regard to jurisdictional claims in published maps and institutional affiliations.

## Supplementary Material

Supporting Information
