# Supplementary material for: An innovative concept of use of redox-active electrolyte in asymmetric capacitor based on MWCNTs/MnO2 and Fe2O3 thin films
Source: Sci Rep. 2016 Dec 16;6:39205. doi: 10.1038/srep39205 (PMC5159809; doi:10.1038/srep39205)
Supplement: Supporting Information [file srep39205-s1.doc]

An innovative concept of use of redox-active electrolyte in asymmetric capacitor based on MWCNTs/MnO_2_ and Fe_2_O_3_ thin films

*Nilesh R. Chodankar^a, e^, Deepak P. Dubal^b^, Abhishek C. Lokhande^c^, Amar M. Patil^a^, Jin H. Kim^c*^, Chandrakant D. Lokhande^d^**

^a^Thin Film Physics Laboratory, Department of Physics, Shivaji University,

Kolhapur, - 416004 (M.S), India

^b^Catalan Institute of Nanoscience and Nanotechnology (ICN2), CSIC and The Barcelona Institute of Science and Technology, Campus UAB, Bellaterra, 08193 Barcelona, Spain

^c^Department of Materials Science and Engineering, Chonnam National University, 300 Yongbong-Dong, Puk-Gu, Gwangju 500-757, South Korea

^d^Centre for Interdisciplinary Studies, D.Y. Patil University, Kolhapur, (M.S.) India

^e^ School of Applied Chemical Engineering, Chonnam National University, Gwangju 500-757, South Korea

**CORRESPONDING AUTHOR FOOTNOTE**

*Prof. Chandrakant D. Lokhande

Tel.: +91 231 2609225, Fax: +91 231 2609233

E-mail address: [l_chandrakant@yahoo.com](mailto:l_chandrakant@yahoo.com)

**CO-CORRESPONDING AUTHOR FOOTNOTE**

^*^Prof. Jin Hyeok Kim

E-mail address: [jinhyeok@chonnam.ac.kr](mailto:jinhyeok@chonnam.ac.kr)

**Supporting Information S1**


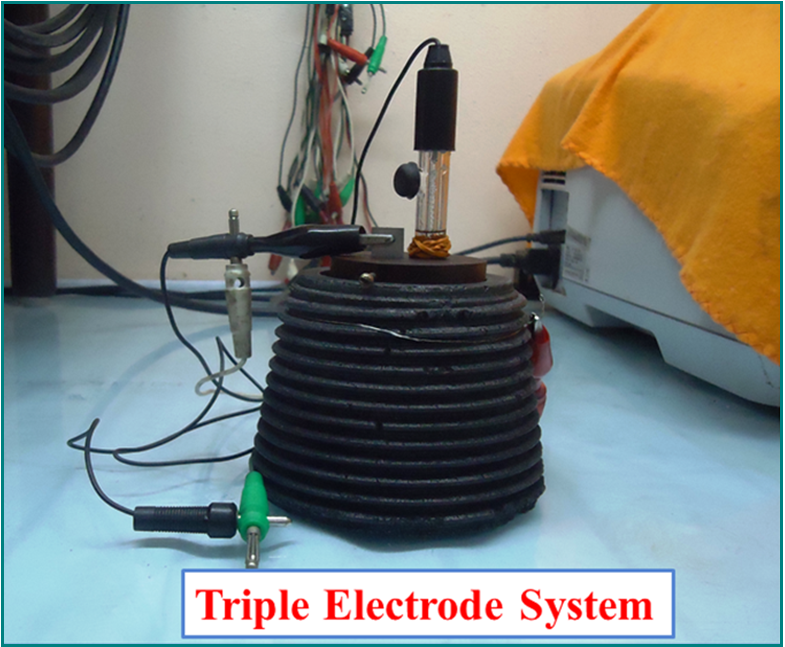


**Fig. S1** the actual three electrodes step used for preparation of MnO_2_@MWCNTs thin films

**Supporting Information S2**

**
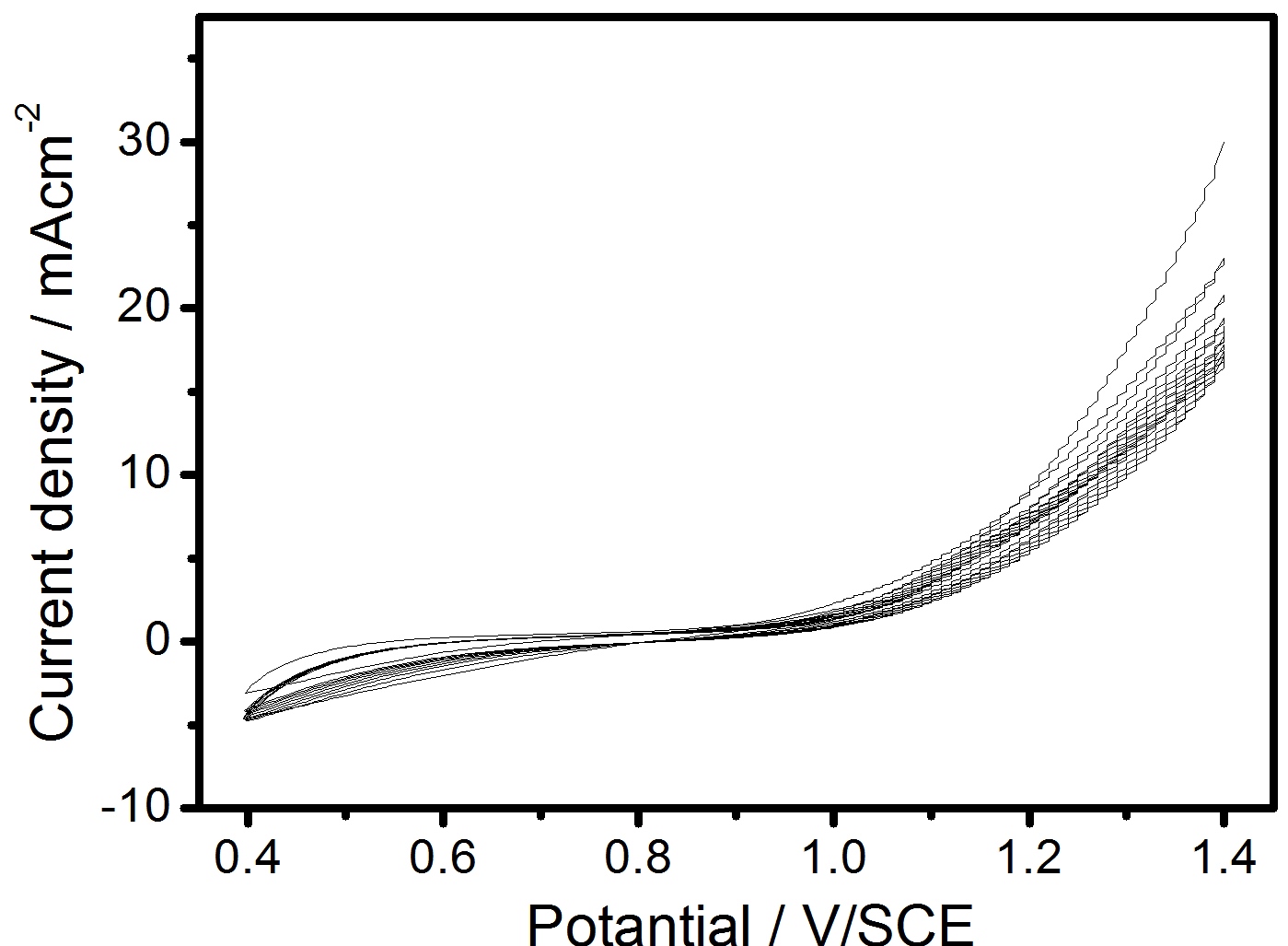
**

**Fig. S2** Cyclic voltammogram of the stainless steel electrode in 0.1 M MnSO_4_ and 0.1 M KOH solution

**Supporting Information S3**


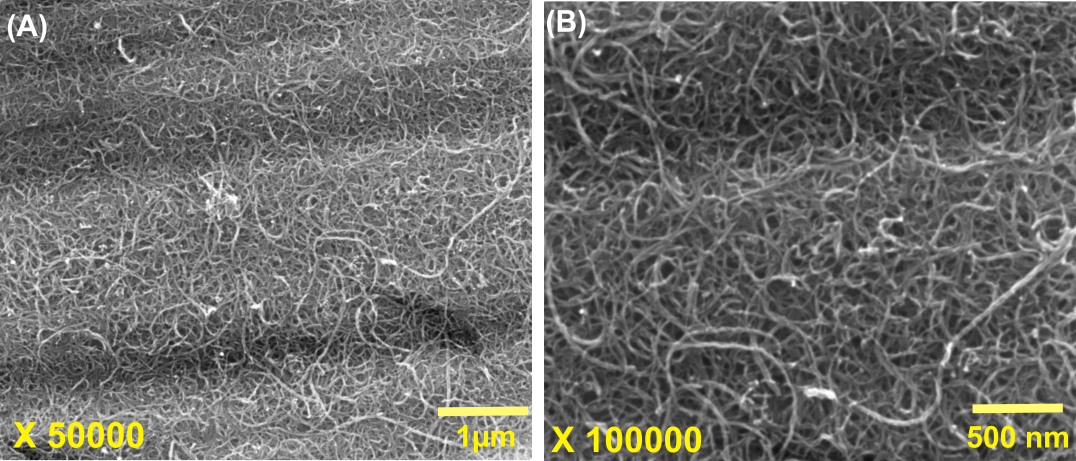


**Fig. S3 (A, B)** the FESEM images of MWCNT thin films at two different magnifications

**Supporting Information S4**

**
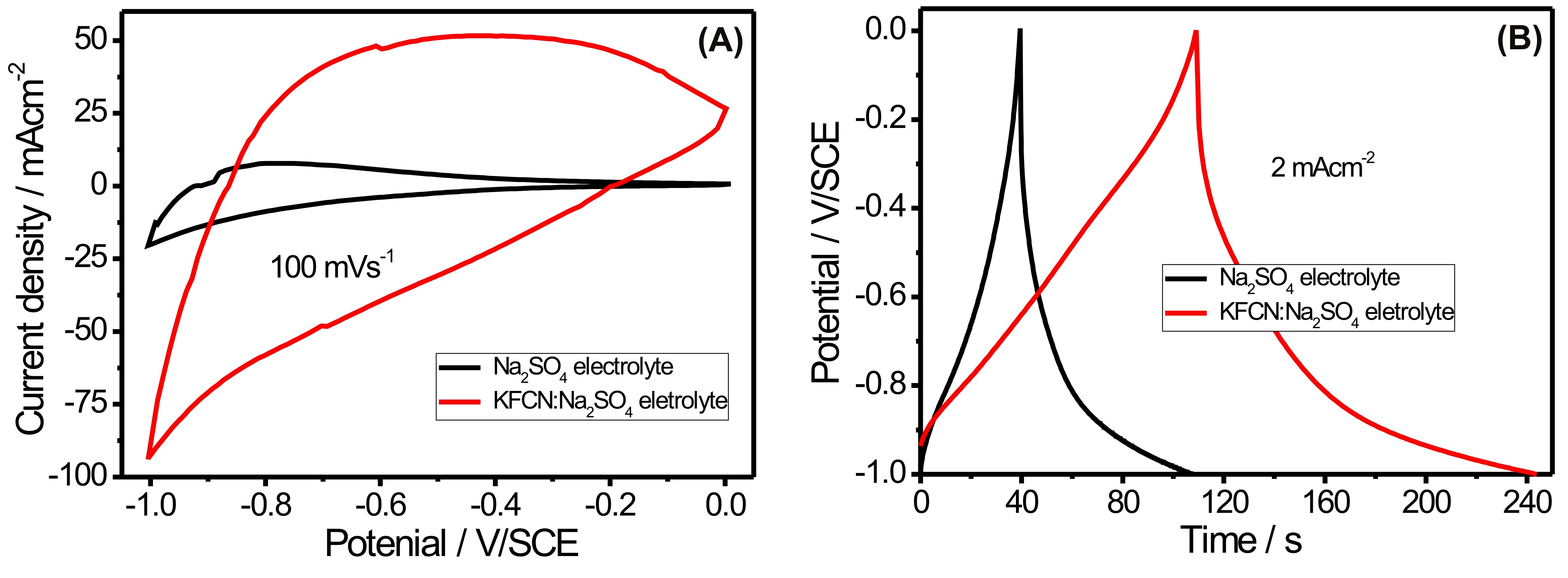
**

**Fig. S4** (A) CV and (B) CD curves of Fe_2_O_3_ thin films at identical condition in Na_2_SO_4_ and KFCN:Na_2_SO_4_ electrolyte

**Supporting Information S5**

**Electrochemical Calculations:**

Using cyclic voltammetry curves, the specific capacitances were calculated by applying following Equation:

$\text{C}_{\text{s}}=\frac{1}{\text{m}\nu\text{(V}_{\text{c}}-\text{V}_{\text{a}}\text{)}}\int_{\text{V}_{\text{a}}}^{\text{V}_{\text{c}}} \text{I(V)dV}$ (S1)

Where, where, C_s_ is the specific capacitance (Fg^-1^), V is the potential scan rate (mVs^-1^), (Vc-Va) is an operational potentional window, I is the current response (mA). Galvanostatic charge–discharge curves were also used to calculate the specific capacitance using following Equation:

$\text{C}_{\text{s}}=\frac{\text{I}_{\text{d}}\times\text{T}_{\text{d}}}{\text{ΔV}\times\text{m}}$ (S2)

Where, C_s_ is specific capacitance, I_d_ is discharge current, T_d_ is the discharge time, ∆V is potential window, and m is the mass of active material. Using the discharge curve, the energy density and power density were calculated by applying following equations:

$\text{E}=\frac{0.5\times\text{C}_{\text{s}}\times\text{(V}_{\text{max}}^{2}-\text{V}_{\text{min}}^{2}\text{)}}{3.6}$ And (S3)

$\text{P}=\frac{\text{E}\times3600}{\text{T}_{\text{d}}}$ (S4)

where, Cs is specific capacitance, T_d_ is discharging time, V_max_ and V_min_ are maximum and minimum potential during charging and discharging cycles.

**Supporting Information S6**

**Table 1**: Comparative chart for MnO_2_ based hybrid electrodes for supercapacitor application

| **Material** | **Electrolyte** | **Specific capacitance** | **Stability** | **Ref.** |
| --- | --- | --- | --- | --- |
| graphene/MnO_2_/CNTs | 0.5 M Na_2_SO_4_ | 380 F g^-1^ | 95% capacitance retention over 3000 cycles | [29] |
| MWCNTs/MnO_2_ | 0.1 M K_2_SO_4_ | 290 F g^-1^ | 88.4% capacitance retention over 1000 cycles | [30] |
| GO-MnO_2_ | 1 M Na_2_SO_4_ | 216 F g^-1^ | 84.1% capacitance retention over 1000 cycles | [31] |
| MnO_2_/MWCNTs | 1 M Na_2_SO_4_ | 255 F g ^-1^ | - | [32] |
| MnO_2_-graphene-CNT | 1.5 M Li_2_SO_4_ | 330.75 F g^-1^ | 90% capacitance retention over 1000 cycles | [33] |
| GO/MnO_2_ | 1 M Na_2_SO_4_ | 327.5 F g^-1^ | 88.26% capacitance retention over 1000 cycles | [34] |
| **MWCNTs/ MnO_2_ nanocomposites** | **1 M Na2SO4 + 0.3 M K_3_[Fe(CN)_6_]** | **1012 F g^-1^** | **92.33%**  **capacitance retention over 3000 cycles** | **Present work** |

**Supporting Information S7**

**Fabrication of the MnO_2_/MWCNTs//Fe_2_O_3_ asymmetric supercapacitor**

In order to fabricate the asymmetric supercapacitor device, we deposited MnO_2_/MWCNTs and Fe_2_O_3_ thin film on flexible stainless steel substrates having an area of 5 cm X 4 cm. Further, the corners of the prepared thin film are sealed with the insulating tape in order to avoid any kind short. The asymmetric supercapacitor device was composed by sandwiching cloth as a separator in between MnO_2_/MWCNTs and Fe_2_O_3_ thin films and further the whole assembly inserted in a small plastic jacket with KFCN:Na_2_SO_4_ electrolyte for the two-electrode system and the jacket is finally sealed. Further the electrochemical measurements are carried out within operating potential of 2.0 V. All electrochemical parameters are calculated using the equations S1 to S4 just by considering the deposited weight of the MnO_2_/MWCNTs and Fe_2_O_3_ on the current collector.

**Supporting Information S8**


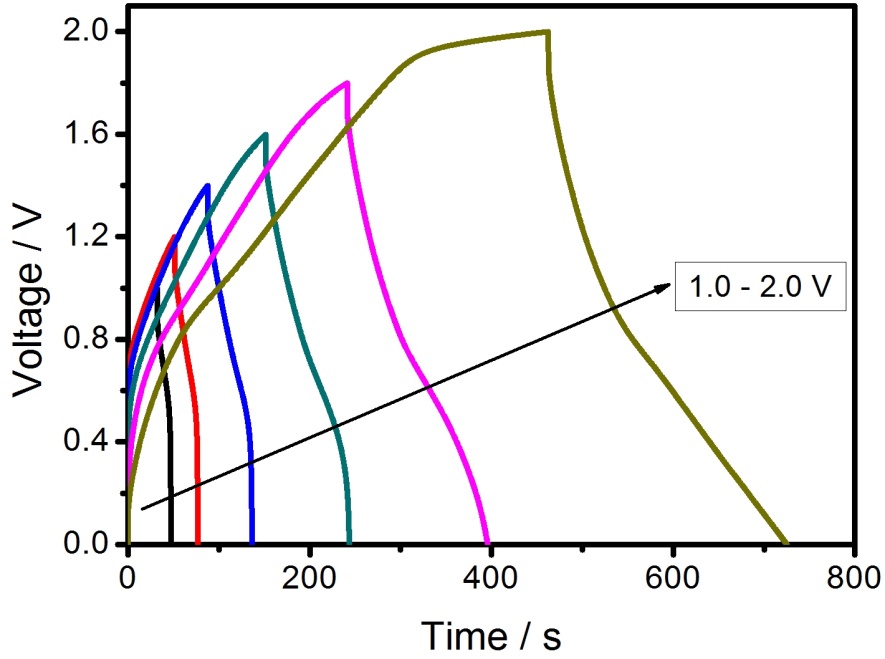


**Fig. S5** the charge discharge curves for asymmetric SCs at different operating potential window

**Supporting Information S9**

**Table 2**: Comparative chart for electrochemical performance of asymmetric SC devices

| **Sr. No.** | **Asymmetric design** | **Electrolyte** | **Operating potential window (V)** | **Specific energy**  **(Wh kg-1)** | **Cycle number** | **Ref.** |
| --- | --- | --- | --- | --- | --- | --- |
| 1. | graphene/MnO_2_ //ACN | Na_2_SO_4_ | 1.8 | 51.1 | 1000 | [35] |
| 2. | RGO/MnO_2_//RGO/MoO_3_ | Na_2_SO_4_ | 2.0 | 42.6 | 1000 | [28] |
| 3. | MnO_2_ //Mesoporous CNT | Na_2_SO_4_ | 2.0 | 47.4 | 1000 | [36] |
| 4. | Graphene/MnO_2_ //Graphene | Na_2_SO_4_ | 2.0 | 30.4 | 1000 | [37] |
| 5. | Graphene/MnO_2_ //ACF | Na_2_SO_4_ | 1.8 | 51.1 | 1000 | [38] |
| 6. | MnO_2_ //Graphene hydrogel | Na_2_SO_4_ | 2.0 | 23.2 | 1000 | [39] |
| 7. | MnO_2_//FeOOH | Li_2_SO_4_ | 1.8 | 24.0 | 2000 | [40] |
| 8. | Carbon spheres/MnO_2_ //Carbon spheres | Na_2_SO_4_ | 2.0 | 22.1 | 1000 | [41] |
| **9** | **MWCNTs/ MnO_2_//Fe_2_O_3_** | **Na_2_SO_4_ + 0.3 M K_3_[Fe(CN)_6_]** | **2.0** | **54.3** | **2000** | **Present study** |

**Supporting Information S10**


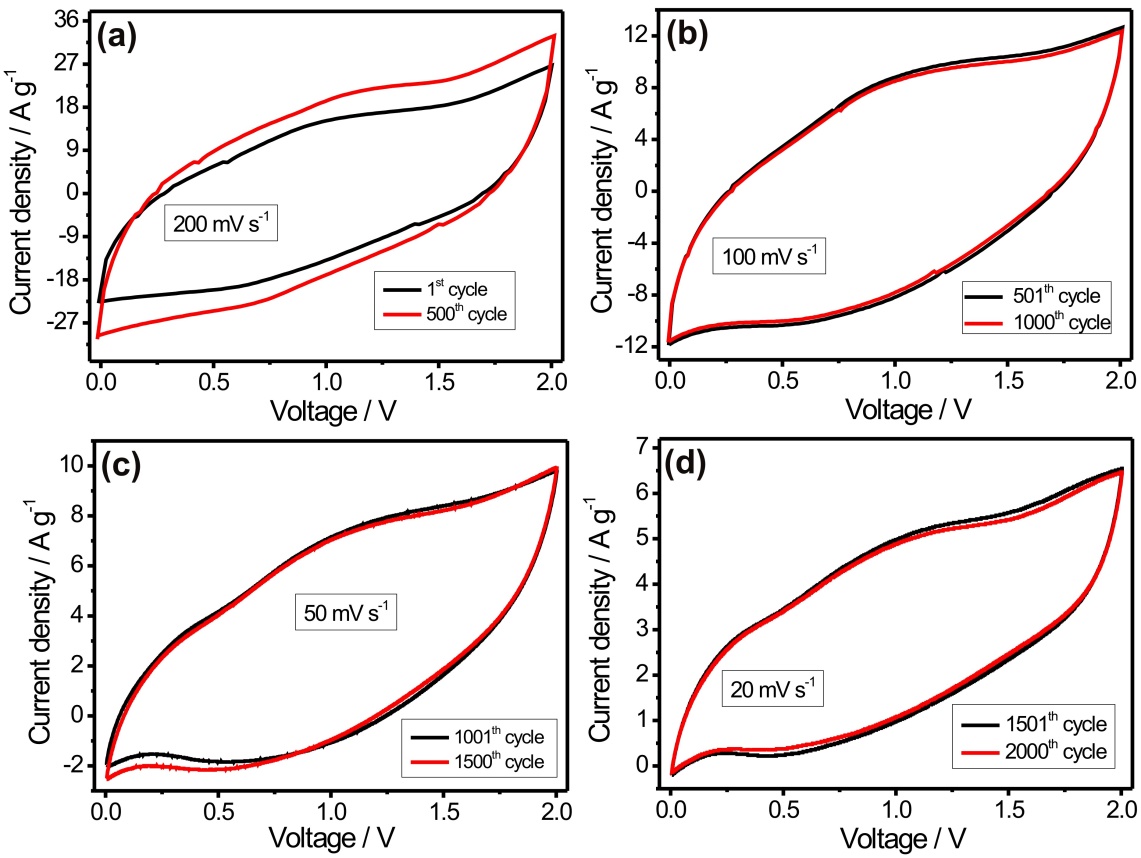


**Fig. 6** Cycling stability of asymmetric SCs at scan rate of (a) 200 mV s^-1^ (b) 100 mV s^-1^ (c) 50 mV s^-1^ and (d) 20 mV s^-1^.

**Supporting Information S10**


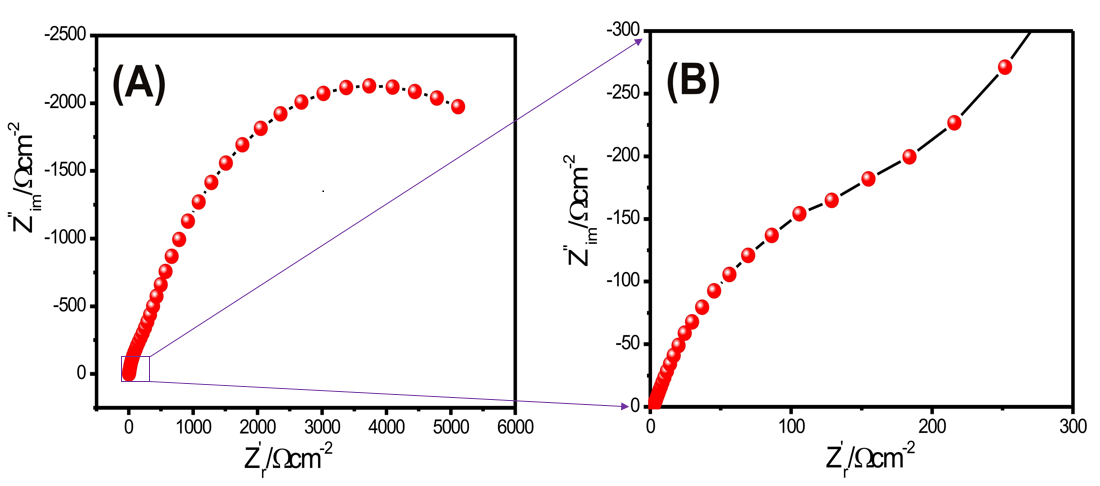


Fig. 7(A) the Nyquist plot for stainless steel electrode and (B) the enlarge view of the Nyquist plot.

**Table 3**: The EIS parameters for stainless steel electrode calculated from the Nyquist plot

|  | ESR (Ωcm^-2^) | Rct (Ωcm^-2^) |
| --- | --- | --- |
| Stainless steel electrode | 3.9 | 198 |
